# Predicting acute kidney injury: current status and future challenges

**DOI:** 10.1007/s40620-017-0416-8

**Published:** 2017-06-17

**Authors:** Simona Pozzoli, Marco Simonini, Paolo Manunta

**Affiliations:** Chair of Nephrology - IRCCS San Raffaele Scientific Institute, Genomics of Renal Diseases and Hypertension Unit, Università Vita Salute San Raffaele, Via Olgettina 60, 20132 Milan, Italy

**Keywords:** Acute kidney injury, Prediction, Biomarkers, Genetics, New OMICs

## Abstract

Acute kidney injury (AKI) is characterized by an acute decline in renal function and is associated to increased mortality rate, hospitalization time, and total health-related costs. The severity of this ‘fearsome’ clinical complication might depend on, or even be worsened by, the late detection of AKI, when the diagnosis is based on the elevation of serum creatinine (SCr). For these reasons, in recent years a great number of new tools, biomarkers and predictive models have been proposed to clinicians in order to improve diagnosis and prevent the development of AKI. The purpose of this narrative paper is to review the current state of the art in prediction and early detection of AKI and outline future challenges.

## Introduction

Acute kidney injury (AKI) is an important health problem. Patients who develop AKI have markedly increased in-hospital mortality and, even if they do survive the acute phase, they still have an increased likelihood of morbidity and mortality over the long term [1–3]. Current treatments focus on avoiding the potential injury due to nephrotoxic drugs or intravenous contrast agents, and on providing supportive care [3]. Theoretically speaking, more specific therapies have been identified in animal models, but their efficacy has not been proven in subsequent human clinical trials; this is because AKI is difficult to identify before loss of organ function sets in, by which time the damage may be irreversible [4]. Therefore, there is growing expectation about the development of biomarkers that can identify AKI in its earliest stage, when interventions could be more successful. In particular, of great interest is the possibility of individual risk stratification for AKI, in order to avoid any unnecessary kidney stress and, if appropriate, to start a preventive treatment [4].

For all these reasons, over the last decade there has been considerable progress made in the discovery and development of new tools, predictive models and biomarkers of AKI; several of them have now been evaluated in different clinical settings [5–7]. Although there is a growing literature on the performance of various biomarkers in clinical studies, information is limited on how these biomarkers could be used by clinicians to manage patients with AKI [5].

The purpose of this narrative paper is to review the current state of the art in prediction and early detection of AKI. This shall be done by describing the most important and promising tools, biomarkers and potential innovations in this field. We will also focus on their real and potential applications in everyday clinical practice.

## New biomarkers of AKI

Accessible markers of AKI can be components of serum or urine. Hopefully, one or more of these biomarkers, either alone or in combination, will prove to be useful in facilitating early diagnosis, guiding targeted interventions and monitoring the disease progression and resolution [8]. The most important and promising biomarkers are summarized in Table 1.

**Table 1:** Overview of the most recent and promising biomarkers for early detection of AKI

Biomarker	Settings studied	Source	Measured from	Used for	Diagnostic accuracy (ROC)
NGAL	Cardiac surgery, ER, hospitalized patients, kidney Tx, sepsis, critically-ill patients	Leukocytes, loop of henle and collecting ducts	Serum plasma	Detection of established AKI, early diagnosis, prognosis	0.53–0.96
			Urine		
Cystatin-C	Hospitalized patients, cardiac surgery	Nucleated cells	Serum plasma	Detection of established AKI, early diagnosis, prognosis	0.79–0.89
			Urine		
KIM-1	Hospitalized patients, cardiac surgery	Proximal tubular cells	Urine	Increased risk of AKI, established AKI, prognosis	0.61–0.78
IL-18	Cardiac surgery, ICU, hospitalized patients, Tx	Monocytes, dendritic cells, macrophages	Urine	Detection of established AKI, early diagnosis, prognosis	0.70–0.95
FABPs	Contrast nephropathy, Sepsis, cardiac surgery, ischemic/reperfusion injury	Hepatocytes, proximal tubular cells	Urine	Detection of established AKI, progression to CKD	0.84–0.96
TIMP-2 and IGFBP7	Major surgery, sepsis, shock, trauma	Tubular epithelial cells	Urine	Detection of established AKI, prognosis	0.76–0.85
EO	Cardiac surgery	Adrenal cells	Plasma	Identification of patients with increased risk of AKI	0.73–0.80

*EO* endogenous ouabain, *ER* emergency room, *FABPs* fatty acid-binding proteins, *ICU* intensive care unit, *IGFBP7* insulin-like growth factor-binding protein 7, IL-18 interleukin-18, KIM-1 kidney injury molecule-1, *NGAL* neutrophil gelatinase-associated lipocalin, *ROC* receiver operating characteristic curve, *TIMP2* tissue inhibitor of metalloproteinases 2, *Tx* transplantation

### Neutrophil gelatinase-associated lipocalin

Human neutrophil gelatinase-associated lipocalin (NGAL) is a 25-kDa protein initially identified bound to gelatinase in specific granules of the neutrophil. NGAL is synthesized during a narrow window of granulocyte maturation in the bone marrow [9], but may also be induced in epithelial cells in the setting of inflammation or malignancy [10]. NGAL should be considered as a marker of tubular damage [11].

NGAL was identified as being one of the seven genes whose expression was upregulated more than tenfold within the first few hours after ischemic renal injury in a mouse model [12]. Although it was shown that exogenous administration of NGAL protects against ischemic kidney injury in mice [13], lipocalin-2 knockout mice do not exhibit increased sensitivity to bilateral renal ischemia/reperfusion injury [14]. NGAL is upregulated and can be detected in the kidney [15] and urine of mice 3 h after cisplatin (20 mg/kg) administration and it has been proposed as an early biomarker for diagnosing AKI [16]. A prospective study of pediatric patients undergoing cardiopulmonary bypass (CPB) for cardiac corrective surgery found urinary NGAL to be a powerful early marker of AKI, preceding any increase in serum creatinine (SCr) by 1–3 days [17]. A similar study of adult patients showed urinary NGAL levels at 1, 3, and 18 h after cardiac surgery to be significantly higher in patients who went on to develop clinically significant AKI [18]. Elevated NGAL levels have also been reported in heart failure, coronary heart disease, and stroke; some studies have shown NGAL to be an independent predictor of major adverse cardiovascular events and mortality [19–21].

NGAL has been one of the most widely studied biomarkers in AKI [22], in particular in the cardio-surgical field [23, 24]. NGAL has been tested in multiple studies that have included a total of more than 4000 patients at risk for AKI due to sepsis, cardiac surgery, exposure to contrast media, or after renal transplantation. In these studies, the average sensitivity and specificity of NGAL ranged from 70 to 80%, upon different king of ARF triggering mechanism (sepsis vs. cardiac surgery). The diagnostic accuracy (receiver operating characteristics, ROC) was among 0.53 and 0.96 [25–28]. Moreover, in a recent extensive meta-analysis of data from 19 studies including >2500 patients, serum and urine NGAL levels were found not only to be diagnostic of AKI, but also able to predict clinical outcomes such as need for dialysis and mortality [21].

NGAL shows the potential to be a simple and powerful biomarker able to provide an early (within a few hours) AKI diagnosis [17], and capable of differentiating between prerenal kidney disease and acute tubular necrosis (ATN) [29]. NGAL tests are available for clinical use in Europe and will eventually be available in North America too, although it is not clear which test (urine vs. plasma sample) provides the best diagnostic performance for AKI. Some authors have suggested that a combination of the two tests might be the best option [25].

### Cystatin-C

Cystatin-C (Cys-C) is a 13-kDa protein that was initially known as interalia γ-trace, post-γ-globulin, and gamma-CSF and is believed to be one of the most important extracellular inhibitors of cysteine proteases. Cys-C is freely filtered by the glomerulus, reabsorbed and catabolized, but not secreted, by the tubules. Over the past decade, serum Cys-C has been extensively studied and found to be a sensitive serum marker of the glomerular filtration rate (GFR) and a stronger predictor than SCr of risk of death and cardiovascular events in older patients [30, 31]. The only rodent study in which Cys-C was measured was in the rat model of end-stage renal disease (ESRD) in which sequential bilateral nephrectomy was carried out 7 days apart. The kinetics of changes in serum Cys-C and creatinine concentrations mimicked the clinical condition [32]. Urinary Cys-C levels have been found to be elevated in individuals with known tubular dysfunction [33, 34]. In addition, Herget-Rosenthal et al. reported that elevated urinary Cys-C levels were highly predictive of poor outcome (i.e. need for renal replacement theory, RRT) in a heterogeneous group of patients with initially nonoliguric AKI [35]. In one prospective study, Cys-C was measured in both the plasma and urine of patients undergoing cardiac surgery. Within the first 6 h urinary values of Cys-C rose predicting AKI, but no change was observed in plasma levels, suggesting that the urinary test might be superior to the plasma assay for the early detection of AKI [36].

When compared with (SCr), Cys-C seems to be less affected by age, gender, and body weight. Serum levels of Cys-C are a more precise indicator of kidney function than SCr levels [37, 38] but seem to be influenced by large doses of corticosteroids, hyperthyroidism, inflammation, hyperbilirubinemia and hypertriglyceridemia [39, 40]. Currently, it is unclear if the value of Cys-C is generalizable to all forms of AKI or is specific to particular populations [41–43].

### Kidney injury molecule-1

Kidney injury molecule-1 (KIM-1) is a type I cell membrane glycoprotein containing a unique six-cysteine immunoglobulin-like domain and a mucin domain in its extracellular region. KIM-1 was initially identified using representational difference analysis on kidneys following ischemia/reperfusion injury: KIM-1 mRNA levels increased more than any other known gene after kidney injury [44]. In preclinical and clinical studies using several mechanistically different models of kidney injury, urinary Kim-1 has been used as an early diagnostic indicator of kidney injury [45, 46]. Several reports have shown that KIM-1 appears to be a very sensitive indicator of AKI in noncardiac surgical patient populations [47], and after cardiac surgery [48]. Han et al. demonstrated marked expression of KIM-1 in kidney biopsy specimens from 6 patients with acute tubular necrosis, and found elevated urinary levels of KIM-1 within 12 h after an initial ischemic renal insult, prior to the appearance of casts in the urine. Moreover, this work showed that increased KIM-1 level was associated with a greater than 12-fold (odds ratio, OR 12.4, 95% confidence interval, CI 1.2–119) risk for the presence of ATN [45]. Liangos et al. studied urinary KIM-1 and *N*-acetyl-β-d-glucosaminidase (NAG) in 201 patients with established AKI and found that elevated levels of urinary KIM-1 and NAG were significantly associated with the clinical composite endpoint of death or dialysis requirement, even after adjustment for disease severity or comorbidity [49].

KIM-1 seems to be very useful in differentiating ATN from other forms of AKI. Furthermore, Koyner et al. also described a predictive preoperative power of KIM-1 in relation to the development of stage 1 and stage 3 AKI; this is probably due to the presence of subclinical proximal tubular injury reflected in increased KIM-1 levels [50].

### Interleukin-18

Interleukin-18 (IL-18) is a cytokine that has been identified as an interferon-γ (IFN-γ)-inducing factor in livers of mice treated with *Propionibacterium acnes* and lipopolysaccharide [51]. The precursor form of IL-18 (24 kDa) is enzymatically cleaved by IL-1β-converting enzyme to produce mature 18-kDa IL-18 protein [52]. Renal IL-18 mRNA levels have been shown to be significantly upregulated following ischemia–reperfusion injury, inflammatory/autoimmune nephritis, and cisplatin-induced nephrotoxicity [53].

Urinary IL-18 levels are elevated in patients with AKI and delayed graft function compared to normal subjects and patients with prerenal azotemia, chronic renal insufficiency, and nephrotic syndrome [54]. IL-18 has been shown to be more elevated in patients with established acute tubular necrosis AKI than in those with prerenal azotemia, urinary tract infection, or chronic kidney disease (CKD) [55, 56]. In particular, in a study of critically-ill adult patients with acute respiratory distress syndrome (ARDS), increased urinary IL-18 was found to be an early marker of AKI, preceding changes in serum creatinine by 1–2 days, and was also an independent predictor of death [56].

### Fatty acid–binding protein

Fatty acid-binding proteins (FABPs) are small (15 kDa) cytoplasmic proteins abundantly expressed in all tissues with active fatty acid metabolism [57]. Two types of FABP have been identified in the human kidney: liver-type FABP (L-FABP) in the proximal tubule and heart-type FABP (H-FABP) in the distal tubule [58, 59]. Free fatty acids (FFAs) in proximal tubules are bound to cytoplasmic FABPs and transported to mitochondria or peroxisomes, where they are metabolized by β-oxidation [60]. Urinary L-FABP has been identified in preclinical and clinical models and has been found to be a potential biomarker in a number of pathologic conditions, including CKD, diabetic nephropathy, IgA nephropathy, and contrast nephropathy. Using human L-FABP (hL-FABP) transgenic mice, it has been demonstrated that protein-overload nephropathy and unilateral ureteral obstruction, two models of renal interstitial injury, are associated with increased expression and urinary excretion of L-FABP [61, 62]. In a clinical study, elevated urinary levels of L-FABP were found to be an independent predictor of AKI (elevation time within 4–24 h) [63]. In both injured models, a less severe tubulointerstitial damage was observed in the transgenic mice when compared with wild-type mice, supporting the notion that L-FABP plays a protective role in the setting of increased renal tubular stress [64]. L-FABP has also been advocated as a potential biomarker for monitoring progression of CKD. Kamijo et al. found increasing L-FABP levels with deterioration of renal function in patients with nondiabetic CKD [62]. In addition, Nakamura et al. have reported that urinary L-FABP may serve as a noninvasive biomarker to discriminate between IgA nephropathy and thin basement membrane disease [65] as well as a potential predictive marker for contrast-induced nephropathy [66]. Although L-FAPB appears to be an attractive candidate biomarker for a number of renal diseases, additional studies are needed to determine the utility of L-FABP in AKI, especially in the setting of ischemia/reperfusion injury, nephrotoxin exposure, and sepsis.

### TIMP-2 and IGFBP7

Tissue inhibitor of metalloproteinases 2 (TIMP2) and insulin-like growth factor-binding protein 7 (IGFBP7) are markers of cellular stress in the early phase of tubular cell injury caused by a wide variety of insults (inflammation, ischemia, oxidative stress, drugs, and toxins) [67–70]. Therefore, both markers are involved in the process of G1 cell-cycle arrest that prevents cells from dividing in the case of damage to the DNA until such damage can be repaired [71]. Importantly, both biomarkers appear as “alarm” proteins for other nearby cells in a paracrine fashion [72, 73]. Two multicenter observational studies were performed in critically-ill patients at risk for AKI [74]. The top two markers from the discovery phase were validated in a second study (Sapphire) and compared to a number of previously described biomarkers. In the discovery phase, 522 adults were enrolled in three distinct cohorts including patients with sepsis, shock, major surgery, and trauma and over 300 markers were examined. In the Sapphire validation study, 744 adult subjects with critical illness and without evidence of AKI (at enrolment) were enrolled; the final analysis cohort was a heterogeneous sample of 728 critically-ill patients (14% with moderate to severe AKI). IGFBP7 and TIMP-2, used together, demonstrated an area under the curve (AUC) for AKI of 0.80 (0.76 and 0.79 alone). Furthermore, combined used of IGFBP7 and TIMP-2 significantly improved risk stratification when added to a 9-variable clinical model.

### Endogenous ouabain

Endogenous ouabain (EO) is a neuroendocrine hormone synthesized in the adrenal cortex [75–77]. EO modulates the activity of Na, K-ATPase and induces signal transduction via sodium-calcium exchange and the Src-dependent pathway [78]. The hypertensive effect of EO is well established in both animal and human models [79–81], as well as its association with organ damage [82, 83]. Furthermore, a rat model of ouabain-induced hypertension exhibited reduced creatinine clearance, proteinuria, and impaired podocyte nephrin expression; thus, elevated EO per se may be a direct cause of podocyte damage. Ouabain-infused rats exhibited a significant reduction of creatinine clearance (−18%, p < 0.02) and an increase in urinary protein excretion (+54%, p < 0.05) compared to controls [84]. The mechanism of the EO effect is likely mediated by changes in cell Ca2^+^ (activation of the Ca^++^-dependent protease calpain [85] with an increase in nephrin protein cleavage [86]) or, via NFkB (active activation of the transcriptional regulator Snail with reduction in nephrin expression [87]).

Recently, a significant association has been reported of preoperative EO levels with adverse renal outcomes in cardiac surgery patients and with mortality in critically-ill patients. In one study [84], elevated preoperative EO levels were associated with a higher incidence of postoperative AKI (20.3 vs. 2.8%, p < 0.001) and ICU stay (2.4 ± 0.59 vs. 1.4 ± 0.38 days, p = 0.014); in a second study [88], the preoperative EO value was added to a different clinical AKI predictive model and resulted in a significant improvement of risk prediction power (AUC of AKI from 0.79 to 0.84; p < 0.0001). Finally, post-operative EO levels were also associated with a higher mortality rate after cardiac surgery [89].

### Other new potential biomarkers

Recently, some new potential biomarkers have been proposed for early determination of AKI in specific conditions. Clusterin [8, 90], osteopontin [91], intestinal trefoil factor (TFF3) [92], glutathione-S-transferase (GST) [93, 94] and pyruvate kinase M2 [95] were associated with the development of drug-induced nephrotoxicity in an in vitro study and animal models focusing on potential new mechanisms of development of renal damage [95]. However, further investigations are needed to confirm these relationships and the potential benefits of these new molecules.

### Transition from AKI to CKD

A potential association has been described between some of the new early biomarkers of AKI and the presence of chronic subclinical kidney damage. In this way, these biomarkers should also be considered markers of progression from AKI to CKD with a prognostic value.

#### NGAL

In a cross-sectional study of 80 non-diabetic patients with CKD stages 2–4, serum NGAL was found to be elevated in those with the most advanced CKD [96]. Moreover, urinary and serum NGAL levels have been noted to be elevated in a wide range of kidney diseases, including diabetic nephropathy, autosomal polycystic kidney disease and IgA nephropathy [97, 98]. NGAL was also identified in an animal model as an active player in kidney disease progression [99].

#### KIM-1

In a retrospective study of patients with non-diabetic proteinuric kidney disease, KIM-1 levels in urine were found to be elevated, but subsequently decreased when patients received treatment with angiotensin-converting enzyme inhibitors or a low-sodium diet [100]. In a recent study of a cohort of patients with type 1 diabetes and proteinuria, serum KIM-1 level at baseline strongly predicted the rate of estimated GFR loss and risk of ESRD during 5–15 years of follow-up [101]. Moreover, in an animal model KIM-1 showed a potential direct role in CKD progression by promoting kidney fibrosis, interstitial kidney inflammation and progressive renal failure with anemia, proteinuria, hypertension, and cardiac hypertrophy [102].

#### L-FABP

In two different studies (on diabetic and non-diabetic patients), urinary L-FABP was found to be more sensitive than proteinuria in predicting the progression of CKD [103, 104].

Cys-C is considered a “functional biomarker” of AKI because it is freely filtered and reabsorbed by the proximal tubule and this process is inhibited in the presence of kidney damage [43]. It has been suggested that Cys-C might better predict the risk of developing CKD, highlighting a state of ‘preclinical’ kidney dysfunction rather than identifying the early phase of AKI [105]. Moreover, other data suggest that Cys-C is modified by age, sex, muscle mass, obesity, smoking status, thyroid function, inflammation, and malignancy. These factors suggest the need for age-specific and sex-specific reference standards [106].

### Ongoing problems with novel biomarkers

Although all these new molecules are promising candidate biomarkers for AKI, they are still rarely used in everyday clinical practice. Even if the primary results are really encouraging, the use of Cys-C, NGAL or other alternative makers of early AKI is still an area of ongoing research [107]. Indeed, recently some authors have shown that there are a couple of “blind spots” in the use of these new markers and, sometimes, the real meaning of the increased levels of NGAL, KIM-1 or other biomarkers is not completely understood [108, 109]. Some authors reported the inability of these new biomarkers to predict AKI with sufficient clinical pertinence to justify the cost of these analyses in routine practice [109, 110]. And some of the biomarkers have still not been evaluated with enough data in very specific populations (infant and elderly) [43, 111].

Furthermore, the assays for detection are not standardized and it is still under discussion whether is better to use urine or plasmatic values. Indeed, Mårtensson et al. proposed that the plasma NGAL level is a closer reflection of systemic inflammation than of the extent of renal injury inflicted [112]. Finally, the presence of these new markers in the urine leads to another problem: the real availability and reliability of these tests in critically-ill patients, where urine output is reduced (or totally absent) and usually forced by drugs. In conclusion, the data in our possession are still inconsistent and additional studies are needed to focus on the cost-effectiveness of earlier detection of AKI with these new compounds compared to creatinine, and to determine whether these biomarkers have complementary value. This is, at least in part, due to the heterogeneity of AKI subtypes, that is a great limit for large population studies in human subjects.

### Sepsis-induced AKI

Sepsis represents the one of the main causes of AKI in developed countries [113, 114]. It is estimated that more than 20% of septic patients may show some degree of AKI, and the mortality rate of this subgroup will increase up to 35% [115, 116]. Although sepsis is one of the most common causes of AKI, the framework for the identification and management of sepsis-induced (or sepsis-associated) acute kidney injury (SI-AKI) has not been well established [114, 117]. Both the severity of the kidney injury and the clinical implications (morbidity and mortality rate) worsen with delayed recognition of the injury itself. Moreover, because no singular effective therapy has been uncovered, early initiation of supportive care is the milestone of therapy (sepsis-associated acute kidney injury). It is easy to understand why early detection is of critical importance in SI-AKI. In fact, traditional urinary indices and biochemistry (such as SCr, FeNa and FeU, urine sodium, etc.) are totally inadequate to delineate subtypes and severity of AKI during sepsis [118–120].

Novel AKI biomarkers already have shown an ability to identify SA-AKI before SCr levels. Plasma and urine NGAL levels were significantly higher in 83 patients with SA-AKI compared to patients with nonseptic AKI [121]. In 150 critically-ill adult patients, urinary NGAL showed significant discrimination for AKI in patients with sepsis (AUC = 0.80) [122] but serum NGAL levels alone showed only a marginal predictive capacity for AKI in children with sepsis (AUC, 0.68). Also KIM-1 was reported to be effective in early (within 3 h of admission) identification of acute kidney dysfunction in a subset of 150 septic patients [123]. In a large multicenter study of critically-ill adults [74], TIMP-2 and IGFB7 showed the best predictive power (AUC = 0.82) in a subset of patients with sepsis. Its AKI prediction power was superior to other novel biomarkers such as NGAL, IL-18, L-FABP and KIM-1.

Finally, some studies have shown an association between SI-AKI and some acute phase proteins or kidney function protein. E-selectin (inflammatory and endothelial activation protein) was associated with AKI in patients after sepsis [124]; microalbuminuria was also able to predict subsequent development of AKI (AUC = 0.86) in an observational cohort study on septic patients [125].

Unfortunately, several studies reported that plasma levels of some molecules (e.g. NGAL and Cys-C) are deeply influenced by the inflammatory state [106, 122, 126, 127]; this could represent a potential limit for the clinical use of these new biomarkers in the presence of systemic inflammation. Simultaneous comparison of plasma and urine levels of biomarkers is mandatory and should represent an effective way to overcome this problem.

## Tools for AKI predicion and severity diagnosis

Recently several tools have been proposed to determine the severity of kidney damage and the long-term patient prognosis after kidney injury has been established [128]. Between these options, the most valuable and promising in terms of cost-effectiveness seem to be the furosemide stress test (FST), the renal functional reserve (RFR) examination and predictive models.

### Furosemide stress test

Koyner et al. [128] recently demonstrated that the 2-h urine output after a standardized high-dose furosemide stress test (FST), 1 mg/kg of furosemide in naive patients or 1.5 mg/kg in those with prior exposure, in clinically euvolemic patients with early AKI has the predictive capacity to identify those with severe and progressive AKI [129]. The area under the ROC curve (AUC) for 2-h urine output after FST was 0.87 for severe AKI (AKIN stage-3) in a subset of 77 patients (p = 0.001). The ideal cutoff for predicting progressive AKI during these first 2 h was a urine volume <200 ml (or <100 ml/h) with a sensitivity of 87.1% and a specificity of 84.1% [129]. These data demonstrate that urine output in the first 2 h after FST outperforms several biomarkers of AKI for the prediction of AKI progression and future need for RRT. Specifically, FST was significantly better than our complete panel of urinary biomarkers at predicting progression to AKIN stage 3. The addition of biomarkers to FST results did not provide any additional benefit. Similarly, FST outperformed all other biomarkers in predicting the end point of receipt of RRT and inpatient death.

### Renal functional reserve test

The concept of renal functional reserve (RFR) was introduced in the 80 s [130]. The renal functional reserve was defined as the ability of the kidney to increase renal plasma flow (RPF) and GFR after a stimulus such as a protein load [131, 132]. GFR is not a fixed function and it may increase in healthy subjects in response to different stimuli (both physiological and pathological); the absence of RFR defines a state of hyper-filtration which seems to be a negative factor for the progression of renal failure [133]. This capacity to increase the level of function depends on an intact nephron mass and describes RFR. In this way, subjects with a reduction in RFR were considered ‘sub-clinical AKI’ with an increased susceptibility of the kidney even in the presence of mild exposure [133]. There is no single validated method/test available to determine RFR in an easy, accurate way and which could be used in clinical practice, although in the past multiple attempts have been made [134–136]. Recently, Sharma and Ronco described a standard protocol for a ‘renal stress test’ (RST) to evaluate RFR using weight-adjusted oral protein loads (1 g/kg) in healthy subjects [137]. This test, performed in 18 healthy volunteers, seems to be very easy and accurate with no clinical risks for the patients. Moreover, Pekkafal and Kara proposed the incorporation of a Doppler resistive index (RI) and pulsatility index (PI) into the assessment of RFR [138]. The RFR assessment should be of particular utility in specific clinical situations (such as determining the status of the kidneys in potential living kidney donors; preparatory evaluation/counseling, etc.) [133].

### Models predicting AKI

**Fig. 1:**
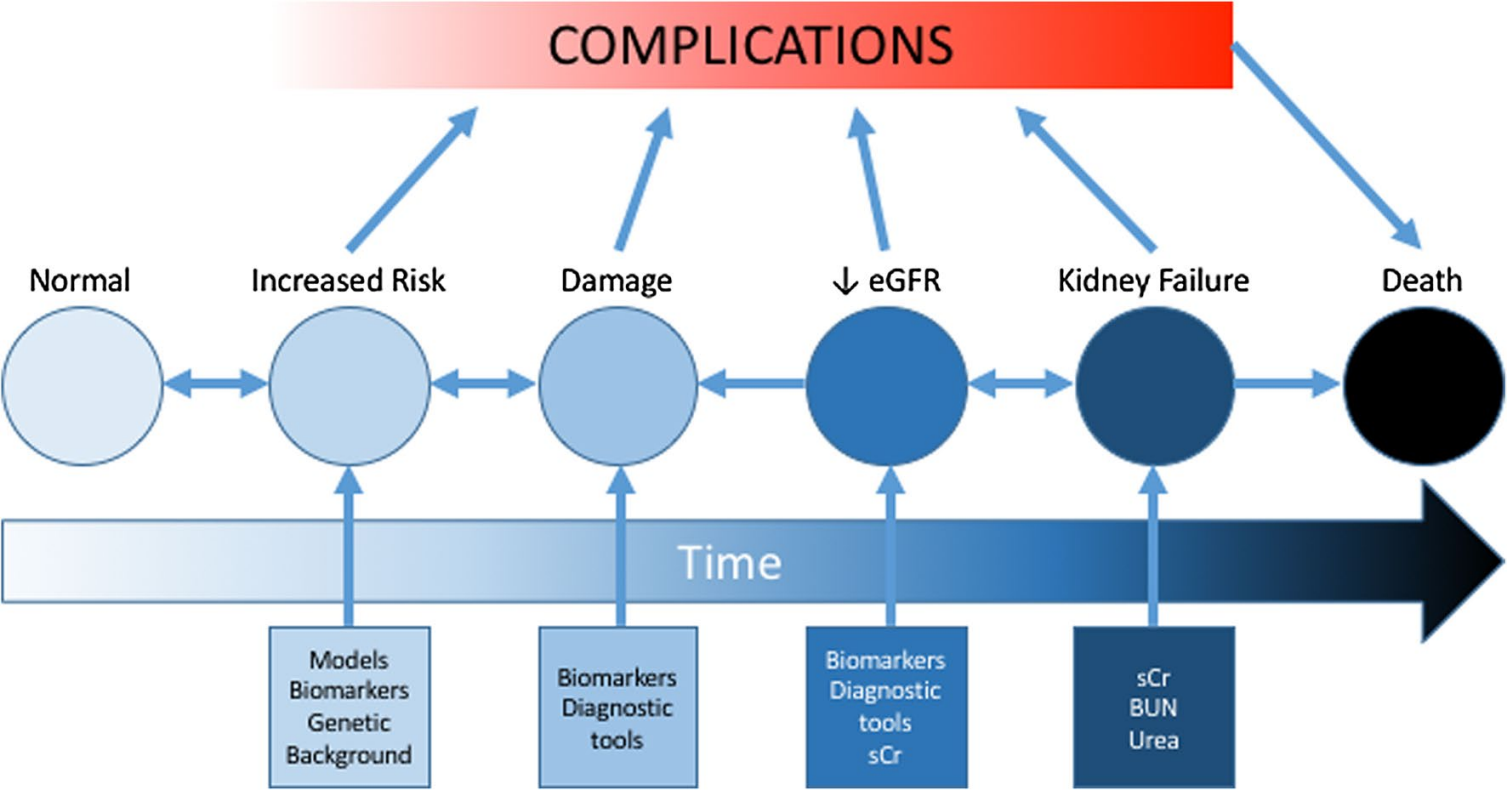
AKI development: distribution of various diagnostic tools across the timeline of the development of acute kidney injury. Clinical predictive models, identification of a favorable genetic background and biomarkers of individual susceptibility (like EO or KIM-1) could be used to identify patients with an increased risk of renal complication. All the other new biomarkers and useful diagnostic tools might be used to determine diagnosis of AKI as early as possible after the damage has occurred

Considering the ongoing issues regarding early AKI detection, it is becoming strategically important to identify subjects with an increased risk of acute renal damage after a therapeutic procedure. This is dramatically true, for example, in post-surgical AKI. More so, neither the new markers of early AKI nor, least of all, the “classic” SCr or blood-urea-nitrogen (BUN) levels are able to identify susceptible patients. All these molecules start to increase in blood and urine when the kidney damage already exists; but it is reported that poor outcomes can be observed just with an increased risk of AKI, even before the kidney damage occurs (see Fig. 1). An accurate, validated prediction model for AKI after cardiac surgery could help in clinical decision making, patient counseling, informed decision making, resource utilization, and preoperative medical optimization [139]. For these reasons, in the last 15 years many new models predicting AKI have been proposed [88, 140–146]. Based simply on good clinical “observation”, these models can predict post-operative AKI with a fairly good power, usually expressed by an AUC between 0.76 and 0.84. Recently, two meta-analyses [7, 147] compared the most important predictive models, showing the strengths and weaknesses of each. The main features of all models are summarized in Table 2.

**Table 2:** Overview of the most important clinical predictive models of post-surgical AKI

Model name	CICSS	Cleveland clinic	STS	SRI	MCSPI	AKICS	NNECDSG	CLIN-RISK
First author	Chertow	Thakar	Mehta	Wijeysundera	Aronson	Palomba	Brown	Simonini
Year of study	1987–1994	1993–2002	2002–2004	1999–2004	1996–2000	2003–2005	2001–2005	2009–2012
Number of patients	42,733	15,838	449,525	10,751	2381	603	8363	802
Outcome (%)	AKI-D (1.1)	AKI-D (1.7)	AKI-D (1.4)	AKI-D (1.3)	AKI-ND (4.8)	AKI-ND (11)	AKI-ND (3)	AKI-ND (9.9)
ROC	0.76	0.81	0.84	0.81	0.84	0.84	0.72	0.79
Validation (ROC)	Yes (0.71–0.78)	Yes (0.66–0.86)	Yes (0.75–0.81)	Yes (0.73–0.79)	Yes^#^ (0.80)	Yes^#^ (0.85)	Yes (0.76)	No
Number of variables	7	13	10	8	8	8	11	8
Demographics		X	X		X	X	X	X
Clinical	X	X	X	X	X	X	X	X
Operation type	X	X	X	X		X		X
Intraoperative					X	X		
Postoperative						X		

*AKI*-*D* AKI requiring dialysis, *AKI-ND* AKI not requiring dialysis, *AKICS* Acute Kidney Injury After Cardiac Surgery Score, *CICSS* Continuous Improvement in Cardiac Surgery Study, *CLIN-RISK* Clinical Risk Score for AKI, *MCSPI* Multicenter Study of Perioperative Ischemia Score, *NNECDSG* Northern New England Cardiovascular Disease Study Group Score, *SRI* simplified renal index, *STS* Society of Thoracic Surgeons Bedside Risk Tool

^#^Only internal validation

The most robust and externally validated models are for AKI requiring dialysis. However, dialysis events are rare (1–2%) and frequently occur several days after the operation, limiting the benefit of application of these scoring systems [7]. More studies are needed to develop and validate scores to predict milder AKI not requiring dialysis, which is very common and contributes to several in-hospital outcomes. Unfortunately, the studies on models with a more sensitive definition of AKI suffer from different definitions of AKI, small cohorts and the lack of external validation. Moreover, in most proposed models the use of intraoperative variables greatly reduces their utility in clinical practice. Indeed, what we would get is a real prediction “*a priori*” of AKI risk, not just a score for post-event outcomes.

Despite all these limitations, the use of clinical prediction models for AKI is currently the only validated strategy available to identify patients with a particularly high risk. Recently, several studies [88] have shown that combining the clinical variables and the new biomarkers could significantly increase the predictive power for the development of AKI compared to the clinical models alone. These results appear more interesting when biomarkers of individual susceptibility are chosen because in this way the *pre-operative* prediction power of the models is preserved.

### Renal angina

A new concept—renal angina—was introduced in 2010 by Goldstein and Chawla [117, 148]. The term ‘angina’ was used in the context of AKI to identify the development of clinical or subclinical renal injury. Renal angina (RA) is not associated to a physical symptomatology (like pain) but is rather a conceptual framework to identify evolving AKI based on the presence of oliguria (for 1 h), any increase in SCr (>0.1 mg/dl), and fluid overload [148–150]. The RA concept has been assessed in one large cohort of critically-ill adult patients with good results: it demonstrated a high sensitivity (92%) associated with the development of AKI, and an extremely high (99%) negative predictive value [151]. Moreover, for a better detection of renal angina, a bedside assessment tool called the Renal Angina Index (RAI) has been developed in the pediatric population. The performance of the RAI in different pediatric cohorts was remarkably consistent, with a risk prediction AUC of 0.74–0.81 [152]. In conclusion, RA probability assessment in AKI appears to have good performance metrics both in children and adults; future research will need to adjust and recalibrate the RA concept, especially in combination with other AKI biomarkers [153].

## Emerging concepts in AKI: genetic susceptibility and new “OMICS” technologies

It is well known in clinical practice that two patients with identical backgrounds and clinical risk factors can react differently to the same insult. Therefore, models using traditional risk factors remain inadequate [142, 154–156]. Furthermore, we are still unable to predict who is going to go on to chronic dialysis and who is going to recover. However, some of these risk factors may be elucidated by two emerging fields: perioperative genomics and new biomarkers derived from the new ‘OMICS’ technologies.

### Genetic susceptibility

In AKI the role of genetic variation as a determinant of both risk and outcome is not well defined [157]. Recently an individual genetic AKI susceptibility has also been proposed [158–160]. Traditional methods of identifying genetic associations are based on multigenerational studies but, by definition, this type of study is not feasible in the field of perioperative medicine. Using association studies, a large number of genetic polymorphisms have been identified that are able to predict different and variable kidney responses in the face of the same kind of injury [157]. In summary, the majority of these high-risk genetic variants are associated to a proinflammatory state, the response to oxidative stress, or alteration of renal vascular response [161]. It is also probable that a patient’s DNA sequence variants have more effect on host repair and regeneration biology than the risk of AKI per se [157].

#### Proinflammatory genes

Usually in postoperative cardiac surgery, the patient’s inflammatory mediators are elevated because CPB, ischemia–reperfusion injury and endotoxemia from general hypoperfusion represent a significant systemic inflammatory trigger [162, 163]. As a consequence, it was supposed that patients who are genetically predisposed to an exaggerated immune response may also be more susceptible to postoperative AKI. Specific polymorphism of IL-6 and tumor necrosis factor (TNF)-α genes were reported to be associated to AKI predisposition (hazard ratio, HR, for TNF-α 2.47, p = 0.04) [155, 159, 164].

#### Renal vascular tone modulators

Polymorphisms of modulators of renal vascular tone have also been proposed as mediators of increased renal risk. These genes include angiotensin-converting enzyme insertion/deletion (ACE I/D), angiotensinogen, angiotensin receptor 1, and endothelial NO synthase [157]. Only a single positive study [165] reported an increased risk in association with ACE D allele (odds ratio, OR, 2.37, p = 0.021).

#### Apolipoprotein E

Polymorphism ε4 apolipoprotein E (APOE), an important regulator of lipoprotein metabolism and immunomodulation, has been associated with a postoperative rise in creatinine in several studies [165, 166] in patients after coronary artery bypass graft (CABG). This finding has not been confirmed in a more consistent multiple studies.

#### Other genes

Oxidative stress genes (like NADPH) and haptoglobin 2–2 polymorphism have been studied as contributors to postoperative risk [167, 168]. Preliminary results showed an association between these gene polymorphisms and AKI onset, dialysis and mortality (OR, respectively, 2.11 and 5.4, p < 0.05).

### Ongoing problems with perioperative genomics

Although several polymorphisms have been investigated, most studies focused on a select number of individual genes in small homogenous sample populations. Overall, the results have been variable and often inconsistent across studies [157]. The lack of robust and reproducible associations is not surprising given the complex, multifactorial nature of perioperative renal injury. In addition, we have a rudimentary understanding of how individual genes may contribute to create a phenotype more prone to develop AKI. Furthermore, none of these studies combined the prognostic information from genetic polymorphisms with existing predictive models. Because of these limitations to association studies, the next step in refining our understanding of at-risk genotypes will require large prospective studies of patients who develop AKI. The ideal model for such clinical studies will continue to be cardiac surgery for several reasons: this represents a high-volume surgical population, the epidemiology of AKI in this setting is well characterized, the timing of the injury is measurable, and improved risk prediction may translate into definable management strategies in the future [161].

### New ‘OMICS’ technologies and AKI

‘Omic’ technologies (e.g. proteomics, metabolomics, exomes, etc.) should give researchers a holistic view of the molecules that are expressed (or overexpressed) in both physiological and pathological conditions [169–171]. These new technologies can be applied not only for a better understanding of normal physiological processes but also in pathological processes where they can play a role in the screening, diagnosis and prognosis as well as in aiding our understanding of the etiology of the diseases. The application of metabolomics, proteomics and functional genomics to evaluate and monitor the presence of acute kidney disease is still under development [172–174]. Validation of these new biomarkers could provide additional tools to detect the onset and severity of kidney injury. Moreover, a significant opportunity exists to integrate metabolomic and proteomic analyses in the study of renal pathophysiology [171, 172]. Several new metabolites [175–178] and exomes [179–181] have been proposed as biomarkers of AKI both in animal and human models. Future work is needed to focus on unambiguous identification of metabolite biomarkers and extensive validation efforts to put these markers to good use for early disease diagnosis in clinical practice [171].

## Conclusion

Acute kidney injury is a very dangerous complication. It is associated with an increased risk of mortality and morbidity, and longer hospital stay, requires additional treatment, and increases the costs of the heath care. This clinical syndrome is characterized by a progressively worsening course, being the consequence of an interplay of different pathophysiologic mechanisms. Several different factors, like hemodynamic or inflammatory status, genetic background and use of nephrotoxic compound, are all involved. Unfortunately, the heterogeneity of AKI subtypes poses a great limit for large population studies in human subjects. In this setting, the use of classic clinical predictive models associated with novel renal biomarkers (both biological and genetic) may well be the only way to refine the methods of treatment and improve the prognosis of patients. Introduction of novel independent biomarkers of AKI into the clinical setting is crucial for earlier diagnosis and improved risk assessment. The purpose of this review was to help clarify the biological basis of new AKI biomarkers that might contribute to improving the early detection or diagnosis of this pathology. But before biomarkers can be advocated for the diagnosis of AKI, further research is needed. Our understanding of how to prevent and manage AKI in an optimal way requires additional effort.

## Abbreviations

AKI: Acute kidney injury
ATN: Acute tubular necrosis
AUC: Area under the curve
BUN: Blood urea nitrogen
CKD: Chronic kidney disease
CYC-C: Cystatin-C
eGFR: Estimated glomerular filtration rate
EO: Endogenous ouabain
FABPs: Fatty acid-binding proteins
FFAs: Free fatty acids
FST: Furosemide stress test
IGFBP7: Insulin-like growth factor-binding protein 7
IL-18: Interleukin-18
KIM-1: Kidney injury molecule-1
NAG: *N*-Acetyl-β-d-glucosaminidase
NGAL: Neutrophil gelatinase-associated lipocalin
OR: Odds ratio
ROC: Receiver operating characteristic curve
RRT: Renal replacement therapy
sCr: Serum creatinine
TIMP2: Tissue inhibitor of metalloproteinases 2
